# Machine learning based prognostic model of Chinese medicine affecting the recurrence and metastasis of I-III stage colorectal cancer: A retrospective study in China

**DOI:** 10.3389/fonc.2022.1044344

**Published:** 2022-11-17

**Authors:** Mo Tang, Lihao Gao, Bin He, Yufei Yang

**Affiliations:** ^1^ Oncology Department, Xiyuan Hospital of China Academy of Chinese Medical Sciences, Beijing, China; ^2^ Smart City Business Unit, Baidu Inc., Beijing, China

**Keywords:** machine learning, traditional Chinese medicine, I-III stage colorectal cancer, recurrence and metastasis, prognostic model

## Abstract

**Background:**

To construct prognostic model of colorectal cancer (CRC) recurrence and metastasis (R&M) with traditional Chinese medicine (TCM) factors based on different machine learning (ML) methods. Aiming to offset the defects in the existing model lacking TCM factors.

**Methods:**

Patients with stage I-III CRC after radical resection were included as the model data set. The training set and the internal verification set were randomly divided at a ratio of 7: 3 by the “set aside method”. The average performance index and 95% confidence interval of the model were calculated by repeating 100 tests. Eight factors were used as predictors of Western medicine. Two types of models were constructed by taking “whether to accept TCM intervention” and “different TCM syndrome types” as TCM predictors. The model was constructed by four ML methods: logistic regression, random forest, Extreme Gradient Boosting (XGBoost) and support vector machine (SVM). The predicted target was whether R&M would occur within 3 years and 5 years after radical surgery. The area under curve (AUC) value and decision curve analysis (DCA) curve were used to evaluate accuracy and utility of the model.

**Results:**

The model data set consisted of 558 patients, of which 317 received TCM intervention after radical resection. The model based on the four ML methods with the TCM factor of “whether to accept TCM intervention” showed good ability in predicting R&M within 3 years and 5 years (AUC value > 0.75), and XGBoost was the best method. The DCA indicated that when the R&M probability in patients was at a certain threshold, the models provided additional clinical benefits. When predicting the R&M probability within 3 years and 5 years in the model with TCM factors of “different TCM syndrome types”, the four methods all showed certain predictive ability (AUC value > 0.70). With the exception of the model constructed by SVM, the other methods provided additional clinical benefits within a certain probability threshold.

**Conclusion:**

The prognostic model based on ML methods shows good accuracy and clinical utility. It can quantify the influence degree of TCM factors on R&M, and provide certain values for clinical decision-making.

## Introduction

With the development of personalized medicine, the prognostic model has received more and more attention in clinical diagnosis and treatment decision-making, disease prognosis management and public resource allocation, and its value is becoming more and more important (1).In clinical practice, Tumor-Node-Metastasis (TNM) staging, which includes three predictors: primary tumor status, regional lymph node involvement, and distant metastasis, is the most widely used prognostic system for colorectal cancer (CRC). However, it ignores some factors that have been proved to be of prognostic value, which results in the prediction system having certain limitations. Therefore, some studies based on Cox proportional hazards regression have improved the accuracy of the clinical prognostic model by including more predictors (2) or adjusting the weight (3) of predictors in TNM stage. However, some studies have found that the models built using these linear methods ignore the time-dependent and nonlinear effects between CRC predictors and prognosis (4), which may cause the phenomenon of “survival paradox”, and result in clinicians being unable to accurately evaluate the prognosis of CRC patients (5, 6).

As a technical branch of computer science, artificial intelligence is good at integrating big data and researchers find it difficult to capture potential patterns and correlations (7). Machine learning (ML) is the main method of realizing artificial intelligence, and aims to develop algorithms that can automatically learn from data (8). It can use complex algorithms to capture large data sets with multi-dimensional variables to obtain high-dimensional and non-linear relationships between clinical features, in order to predict data-driven results (9). The application of data-driven ML methods in prognostic models has broad prospects, and has affected the recognition of the value of medical big data in the field of clinical research (10, 11). At present, the clinical prognostic model based on ML methods has shown high accuracy in many diseases such as lung cancer (12), breast cancer (13) and acute coronary syndrome (14).

Patients with stage I–III CRC are encouraged to follow the guidelines for regular follow-up after routine treatment in Western medicine to monitor tumor R&M status (15). However, some studies have shown that the follow-up strategy under the unified standard is not suitable for all patients, and the survival benefits of individuals are different (16, 17). Therefore, formulating individualized adjuvant treatment and follow-up strategies for patients is an urgent problem which needs to be solved (18). At present, many studies have constructed and developed prognostic models for stage I-III CRC (19, 20). Our research team also used the ML method to construct a prognostic model based on patients with stage I-III colon cancer, and the model showed good predictive ability (21). However, there are no reports on the development of a CRC prognostic model containing traditional Chinese medicine (TCM) factors (such as syndrome type and duration of taking TCM). Promoting the complementary advantages of TCM and Western medicine is a health care model with Chinese characteristics (22). Even though there is a large amount of evidence that TCM intervention is associated with a lower recurrence and metastasis (R&M) rate of CRC, evidence based on population samples is difficult to effectively apply to individualized medical care.

**Figure 1:**
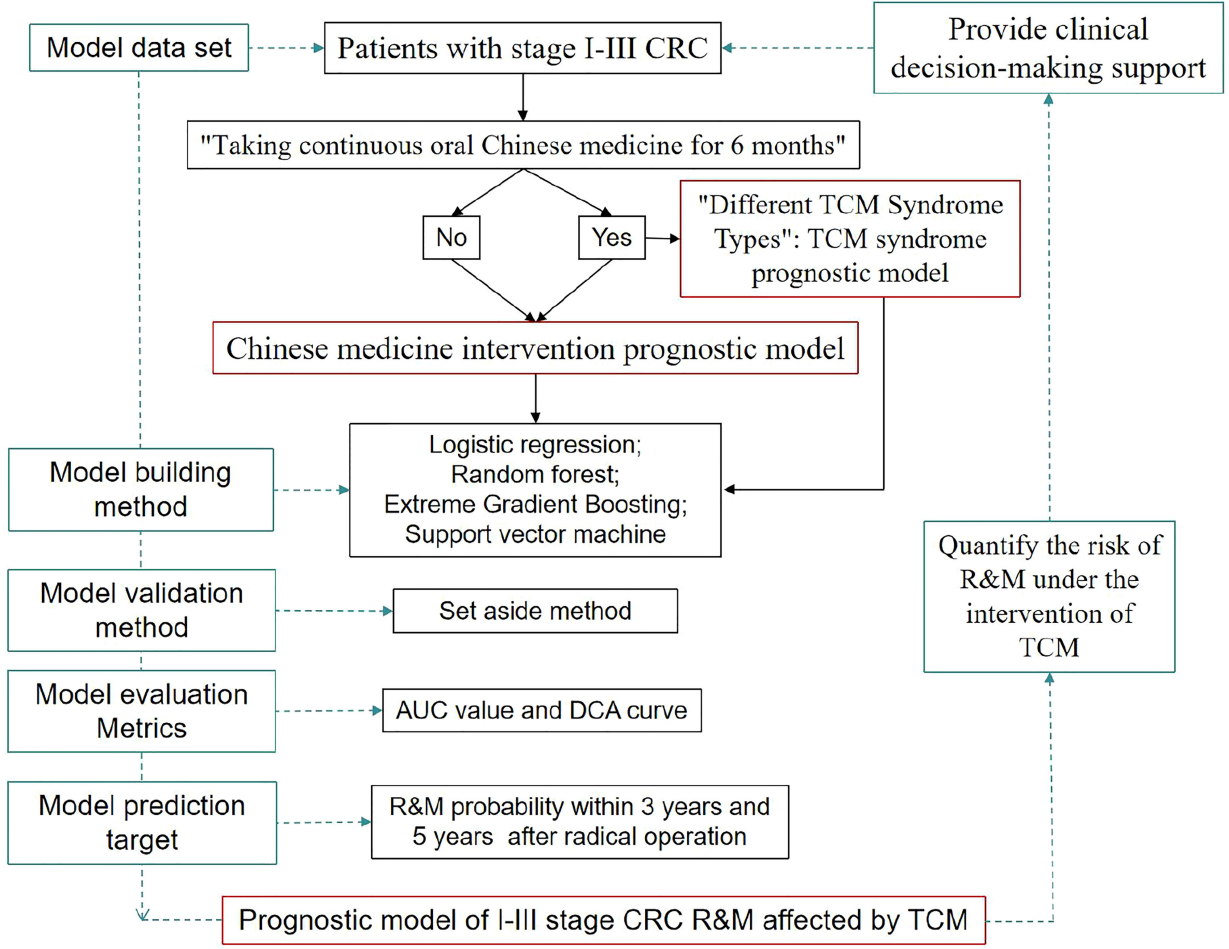
Flowchart of study profile.

Therefore, based on four different ML methods and integrating Western medicine predictors, this study has built a CRC prognostic model with TCM factors for the first time, with a view to quantifying the benefit of patients receiving TCM intervention and the impact of different Chinese medicine syndromes on the R&M of patients, and assisting clinical decision-making. More specifically, based on different ML methods, the patients after radical resection of stage I-III CRC were included as model data sets, and the “prognostic model of CRC R&M whether to take oral Chinese medicine” (hereafter referred to as the “Chinese medicine intervention prognostic model”) and the “prognostic model of CRC R&M with different TCM syndromes” (hereafter referred to as the “TCM syndrome prognostic model”) were constructed, and the model performance under different ML methods was compared and analyzed. The flow diagram of study was shown in **Figure 1**.

## Methods

### Study data

Patients with stage I-III CRC treated in Xiyuan Hospital of Chinese Academy of Chinese Medical Sciences and Beijing Cancer Hospital were included as model data sets, and screening and data collection were conducted according to the following inclusion and exclusion criteria. Inclusion criteria: (1) Gender was not limited, and patient age was 18 years or older; (2) Patients with stage I–III colorectal adenocarcinoma with a definite pathological diagnosis; (3) 3 years or more after radical surgery. Exclusion criteria: (1) Previous or combined history of other incurable malignancies; (2) Patients with R&M within 6 months after radical surgery were considered to have concurrent metastasis (23, 24), and were excluded. The patient’s outpatient medical record (paper version or electronic file) was retained complete and traceable. Through retrospective case observation, the relevant information on patients was collected. The model data set was randomly divided into the training set and the internal verification set according to a 7:3 ratio by the “set aside method”, and the average performance index and 95% confidence interval of the model were further calculated by repeating 100 tests.

### Model predictors

The Western medicine predictors were determined according to the predictor in the reported prognostic model (25) and the actual record of the case data in this study. The details are as follows: (1) Age at diagnosis; (2) primary tumor (T stage); (3) number of positive lymph nodes; (4) whether the number of detected lymph nodes was less than 12; (5) whether there was lymphatic, vascular and nerve invasion; (6) tumor site; (7) microsatellite status; and (8) whether adjuvant chemotherapy was administered. Among them, “age at diagnosis” was used to find the best cutoff value with X-tile software (version 3.6.1) (26), and convert the continuous variable into a dichotomous variable. The TCM predictors in the “Chinese medicine intervention prognostic model” were “whether or not to take Chinese medicine”, and the continuous variables were discretized according to whether or not to take oral Chinese medicine for more than 6 months. The TCM predictor in the “TCM syndrome prognostic model” was “TCM syndrome type”, which was divided into spleen qi deficiency, kidney yin deficiency, spleen and kidney deficiency and non-spleen deficiency and kidney deficiency according to the guiding principles for clinical research of new Chinese materials (27) and the national standard of the people’s Republic of China “TCM clinical diagnosis and treatment terminology syndrome part” (GB/T l6751.2-1997) (28). Details were described in **Table 1**.

**Table 1:** Description of model predictors.

Predictors		Description and assignment	type
Western medicine	Age at diagnosis	According to the best cut-off value, the variables were divided into two categories	dispersed
	T stage	T1=1; T2=2; T3=3; T4a=4; T4b=5	dispersed
	Number of positive lymph nodes	quantity	continuity
	Whether the number of detected lymph nodes is less than 12	Yes = 1; No = 0	dispersed
	Whether there is lymphatic, vascular and nerve invasion	Yes = 1; None = 0	dispersed
	Tumor site	Colon = 1; Rectum = 2	dispersed
	Whether the microsatellite status is stable	Yes = 0; No = 1; Not checked = 2	dispersed
	Whether adjuvant chemotherapy	Yes = 1; No = 0	dispersed
Chinese medicine	“Chinese medicine intervention prognosis model”: whether to take Chinese Medicine	TCM< 6 months = 0; Chinese medicine ≥ 6 months = 1	dispersed
	“TCM syndrome prognostic model”: TCM syndrome type	Spleen qi deficiency = 1; Kidney yin deficiency = 2; Spleen and kidney deficiency = 3; Non spleen deficiency and kidney deficiency = 4	dispersed

### Model prediction target

The prognostic outcome was coded as 0 or 1, respectively, indicating that the patient had no R&M (0) or had R&M (1) at a certain time point. The results of a meta-analysis showed that 80% of patients had early and mid-stage CRC R&M in the first three years after radical surgery (29), and 95% had R&M within five years after radical surgery (30). If the patient had no tumor progression after the 5-year follow-up following radical surgery, CRC was clinically cured (31). Therefore, in this study, the probability of R&M within 3 years and 5 years after radical surgery were respectively used as prediction targets to construct prognostic models.

### Model methods

The prognostic model constructed in this study belongs to a dichotomous problem. Considering the possible nonlinear relationship between the predictors and the prediction target, 4 linear and nonlinear methods were selected for a comparative study. They were logistic regression (LR) (32), random forest (RF) (33), extreme gradient boosting (XGBoost) (34) and support vector machines (SVM) (35). All ML methods were implemented through the Python open source code library (36) “Scikit-learn” (37) and “XGBoost” (34).

### Model evaluation indicators

In this study, the area under curve (AUC) value was selected to evaluate the accuracy of the model. AUC value is the area under the receiver operating characteristic (ROC) curve. The abscissa of the ROC curve is the false positive rate, representing the specificity of the model. The ordinate represents the true positive rate and the sensitivity of the model (38). If the data set used to build the model was limited, the ROC curve will be stepped. The value range of AUC was [0, 1]. The larger the AUC value, the stronger the classification ability. It is generally believed that when the AUC value is ≥ 0.7, the model has good discrimination capacity (39). Decision curve analysis (DCA) can consider the clinical utility of the model, and then integrate the preferences of patients or decision makers into the analysis, which can meet the actual needs of clinical decision-making (40). The abscissa is the threshold probability and the ordinate is the net benefit rat (41). It has two baselines (reference lines), representing the two extreme cases where all samples are predicted to be negative or positive. A model with clinical utility should ensure that its DCA curve is located outside the two reference lines to ensure that it is within the probability range of a certain threshold, and the net income predicted by the model is higher than the two extreme cases. For the DCA curve constructed by various methods, the farther it is from the two reference lines, the higher its application value (42). We also reported precision, recall, accuracy and F1 score to evaluate models performance.

## Results

### Baseline characteristics

A total of 558 outpatients met the inclusion criteria, and of these, 317 patients received Chinese medicine intervention after radical resection of CRC as a sample set of the “TCM syndrome prognostic model”. Of the 558 patients, 181 had R&M within 5 years after radical surgery, accounting for 32.4%, and 377 had no R&M, accounting for 67.6%. **Table 2** shows the basic characteristics of the patients in the model data set. The median age at diagnosis was 58 years, and the optimal cutoff value calculated according to X-tile was 49 years. In terms of T stage, T3 accounted for the largest proportion (59.9%). The median number of positive lymph nodes was 1, ranging from 0 to 24. Overall, 31.2% of the patients had less than 12 positive lymph nodes, 28.7% had vascular, lymphatic or nerve invasion; and 52.5% had stable microsatellite status. The number of patients with a tumor in the colon or rectum was not significantly different, accounting for 52.6% and 47.4% respectively. Overall, 76.5% of the patients received postoperative adjuvant chemotherapy.

**Table 2:** characteristics of basic data of patients in model data set.

Predictors	Number of cases (n = 558)	Proportion
Age at diagnosis,years		
18-49	120	21.5%
> 49	438	78.5%
T stage		
T1	15	2.7%
T2	79	14.2%
T3	334	59.9%
T4a	103	18.5%
T4b	27	4.8%
Number of positive lymph nodes		
Median (min, max)	1(0, 24)	
Lymph nodes is less than 12		
yes	174	31.2%
no	384	68.8%
lymphatic, vascular and nerve invasion		
yes	160	28.7%
no	398	71.3%
Tumor site		
colon	292	52.3%
rectum	266	47.7%
Whether the microsatellite status is stable		
yes	293	52.5%
no	26	4.7%
Unknown	239	42.8%
Adjuvant chemotherapy		
yes	427	76.5%
no	131	23.5%
Chines medicine intervention		
yes	317	56.8%
no	241	43.2%
R&M within 5 years		
yes	181	32.4%
no	377	67.6%

### Model parameter

The four selected ML models were trained with weighted category weights, which were the reciprocal of the number of positive and negative samples. Two R&M scenarios were used, namely, within 3 years and within 5 years. Each specific model used the same parameter configuration. The main parameters of the model were configured as follows: LR used the L2 regularization method, and the penalty coefficient was 20. The number of trees in the RF was 500, the maximum depth of the tree 3, the maximum number of features 3 and the minimum number of leaf nodes 3. The number of base learners of XGBoost was 10000, the maximum depth of the tree 4, the maximum number of features 3 and the learning rate 0.0001. Finally, the kernel function of SVM was radial basis function.

### Chinese medicine intervention prognostic model

**Figure 2:**
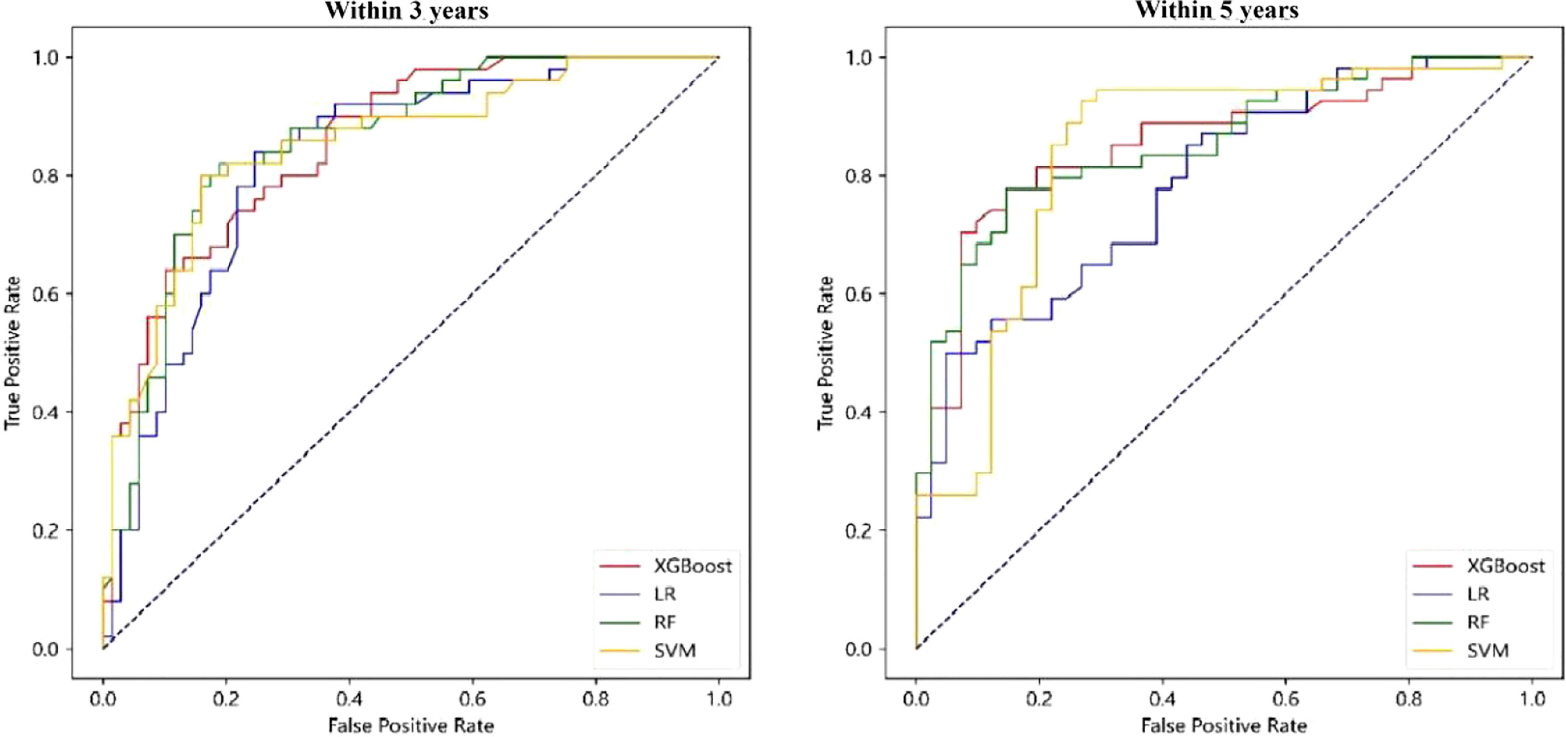
ROC curve of “Chinese medicine intervention prognostic model”.

Based on LR, RF, XGBoost and SVM, respectively, the “Chinese medicine intervention prognostic model” was constructed. The training set and validation set were randomly split and the model performance was calculated. A total of 100 trials were conducted. The values in brackets are AUC values of 95% and 5% percentiles as 95% CI. The results showed that the AUC indices of the four models were 0.83 (0.77, 0.89), 0.86 (0.82, 0.91), 0.86 (0.82, 0.91) and 0.85 (0.79, 0.90), respectively. For the R&M probability model within 5 years, the AUC indices of the four models were 0.79 (0.72, 0.86), 0.83 (0.76, 0.90), 0.85 (0.78, 0.91) and 0.85 (0.78, 0.92), respectively. The model showed good prediction accuracy within 3 years and within 5 years, while the model based on RF and XGBoost methods performed better (see **Table 3** and **Figure 2** for visual comparison). Specifific metrics of precision, accuracy, recall and F1-score were reported in **Supplementary material 1**.

**Table 3:** AUC value comparison of Chinese medicine intervention prognostic model.

Method	AUC value (95%CI)	
	Within 3 years	Within 5 years
LR	0.83 (0.77, 0.89)	0.79 (0.72, 0.86)
RF	0.86 (0.82, 0.91)	0.85 (0.78, 0.91)
XGBoost	0.86 (0.82, 0.91)	0.85 (0.78, 0.92)
SVM	0.85 (0.79, 0.90)	0.83 (0.76, 0.90)

**Figure 3:**
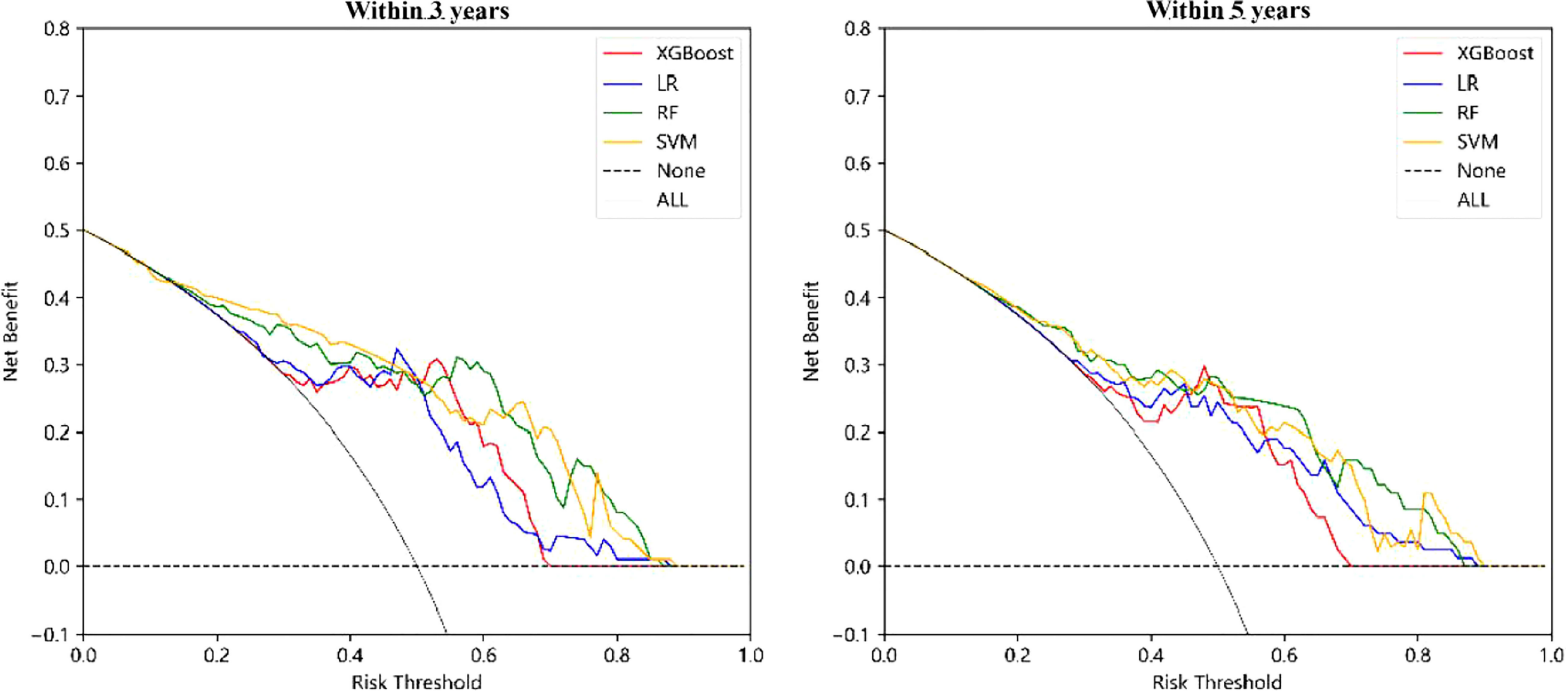
DCA curve of “Chinese medicine intervention prognostic model”.

When constructing the DCA curve, the proportion of positive and negative samples in the validation set was selected as 1:1 in this study to facilitate a comparative analysis. The DCA curve of the “Chinese medicine intervention prognostic model” is shown in **Figure 3**, in which the black dotted line parallel to the X axis and the black smooth curve with a starting value of 0.5 are two reference lines. It can be seen from the figure that when the R&M probability within 3 years and 5 years was taken as the prediction target, the four models were all outside the two reference lines, which indicated that the models constructed by the four methods corresponded to the positive net income within a certain probability threshold range and provided additional clinical benefits. In contrast, the threshold range of the RF and SVM models was larger. In the case of R&M within 3 years and 5 years, they reached approximately 0.2–0.9, respectively, and the XGBoost model was stable at approximately 0.3–0.7.

### TCM syndrome prognostic model

**Figure 4:**
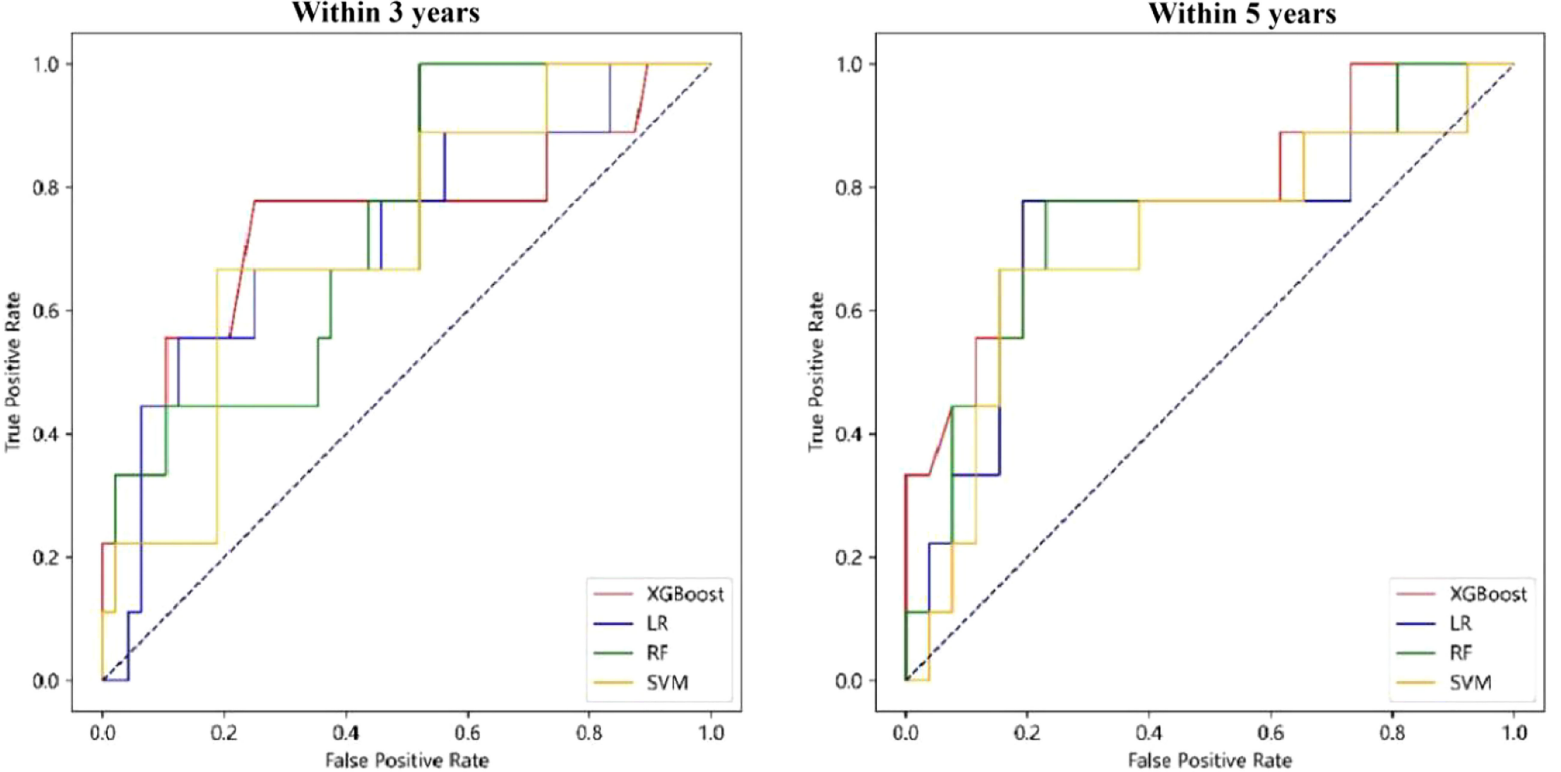
ROC curve of “TCM syndrome prognostic model”.

Similar to the construction method of the “Chinese medicine prognostic model”, the “TCM syndrome prognostic model” was created. The results in **Figure 4** and **Table 4**, show that the AUC values of LR, RF, XGBoost and SVM were 0.72 (0.59, 0.84), 0.74 (0.63, 0.85), 0.75 (0.64, 0.87) and 0.71 (0.59, 0.81), respectively, for the prognostic model within 3 years. XGBoost and RF were relatively better. For the prognostic model within 5 years, the model constructed by XGBoost showed the best performance, with an AUC value of 0.79 (0.65, 0.92), followed by RF, LR and SVM, with AUC values of 0.74 (0.59, 0.88), 0.73 (0.59, 0.86) and 0.71 (0.58, 0.83), respectively (see **Table 4** and **Figure 4** for details). Specifific metrics of precision, accuracy, recall and F1-score were reported in **Supplementary material 1**.

**Table 4:** AUC value comparison of “TCM syndrome prognostic model”.

Method	AUC value (95%CI)	
	Within 3 years	Within 5 years
LR	0.72 (0.59, 0.84)	0.73 (0.59, 0.86)
RF	0.74 (0.63, 0.85)	0.74 (0.59, 0.88)
XGBoost	0.75 (0.64, 0.87)	0.79 (0.65, 0.92)
SVM	0.71 (0.59, 0.81)	0.71 (0.58, 0.83)

**Figure 5:**
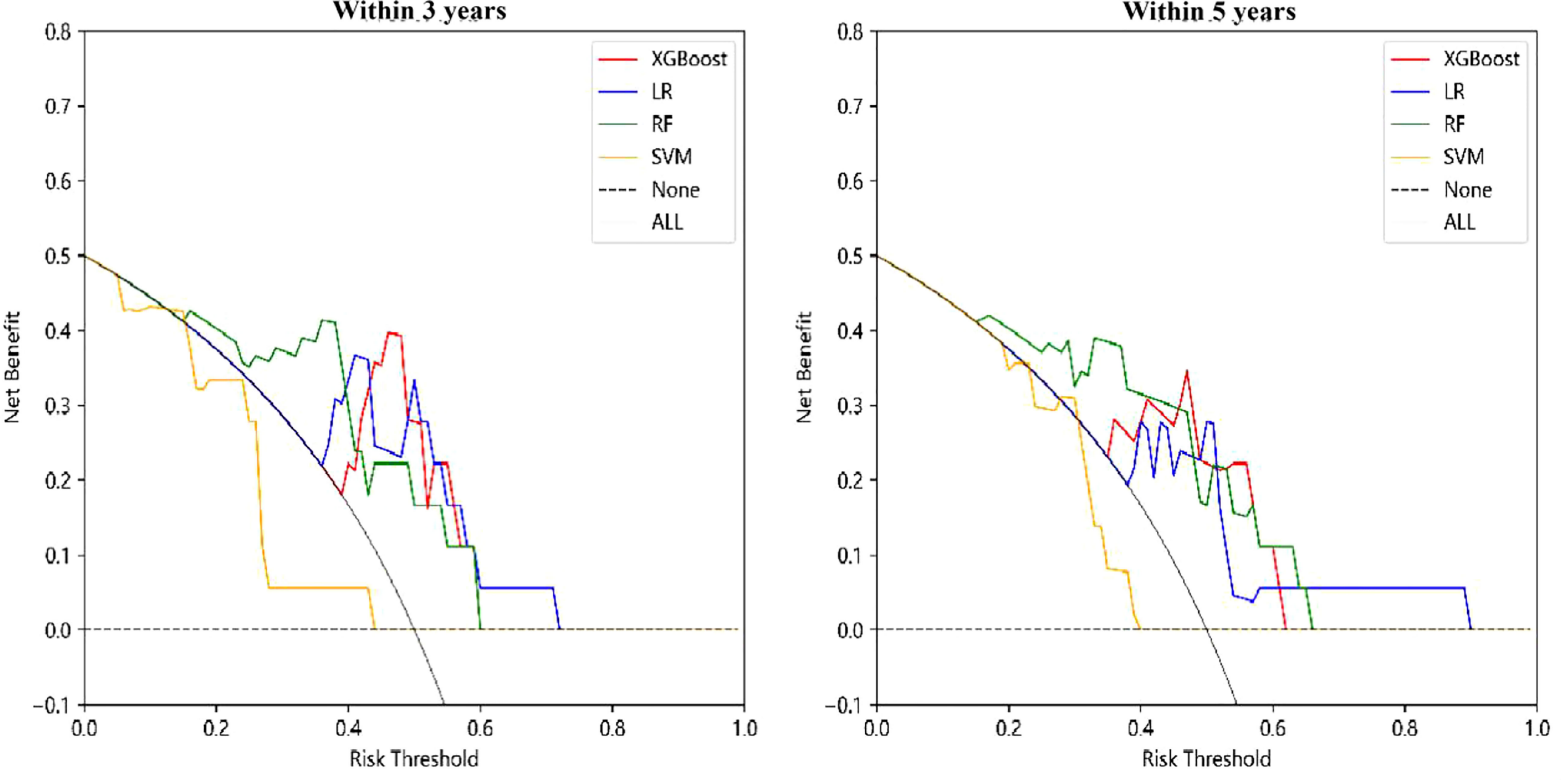
DCA curve of “TCM syndrome prognostic model”.

The DCA curve of the “TCM syndrome prognostic model” is shown in **Figure 5**, in which the black dotted line parallel to the X axis and the black smooth curve with a starting value of 0.5 are two reference lines. It can be seen from the figure that LR, RF and XGBoost all corresponded to positive net income under a certain range of thresholds. The threshold range of net income of RF was the largest, at approximately 0.2–0.7. For the R&M prognostic models within 3 years and 5 years, SVM had no positive net income, and the other three methods had net positive income within a certain probability threshold range. Although the specific range was different, in general, the probability threshold range of RF and LR was larger, and the difference between the upper and lower limits of the threshold was more than 0.4.

### Application of the model

The model constructed in this study can predict the probability of R&M in new patients within 3 years and 5 years. Taking the “Chinese intervention prognostic model” for predicting R&M within 5 years constructed by XGBoost as an example, the model was introduced. The trained model was *f_xgb_* and the characteristic (predictor) of a patient was *X*, and the corresponding values were: {age at diagnosis = 1, T stage = 2, number of positive lymph nodes = 6, number of detected lymph nodes< 12 = 0, presence of lymphatic vessels, blood vessels and nerve invasion = 1, tumor site = 1, microsatellite status = 0, whether adjuvant chemotherapy was administered = 0, and whether taking Chinese medicine = 0}; then the probability of R&M of the patient within 5 years was as follows:


$$P_{i}=f_{xgb}\left(X\right)$$

The actual calculation result was 0.49. Similarly, the probability of R&M in patients within 3 years and 5 years can be obtained by the “TCM syndrome prognostic model” and different ML models.

Furthermore, assuming that the patient takes Chinese medicine, that is, the characteristic value of “whether or not to take Chinese medicine” in *X* was changed to 1, and the new characteristic was marked as *X*’, the probability of R&M in the patient within 5 years when taking Chinese medicine can be estimated as follows:


$$P_{i}^{'}=f_{xgb}\left(X'\right)$$

The actual calculation result was 0.34, so the effect of the patient taking Chinese medicine on R&M was:


$$\Delta P_{i}=P_{i}^{'}-P_{i}$$

The actual calculation result was -0.15, that is, the R&M rate of the patient within 5 years was reduced by 15 percentage points.

**Figure 6:**
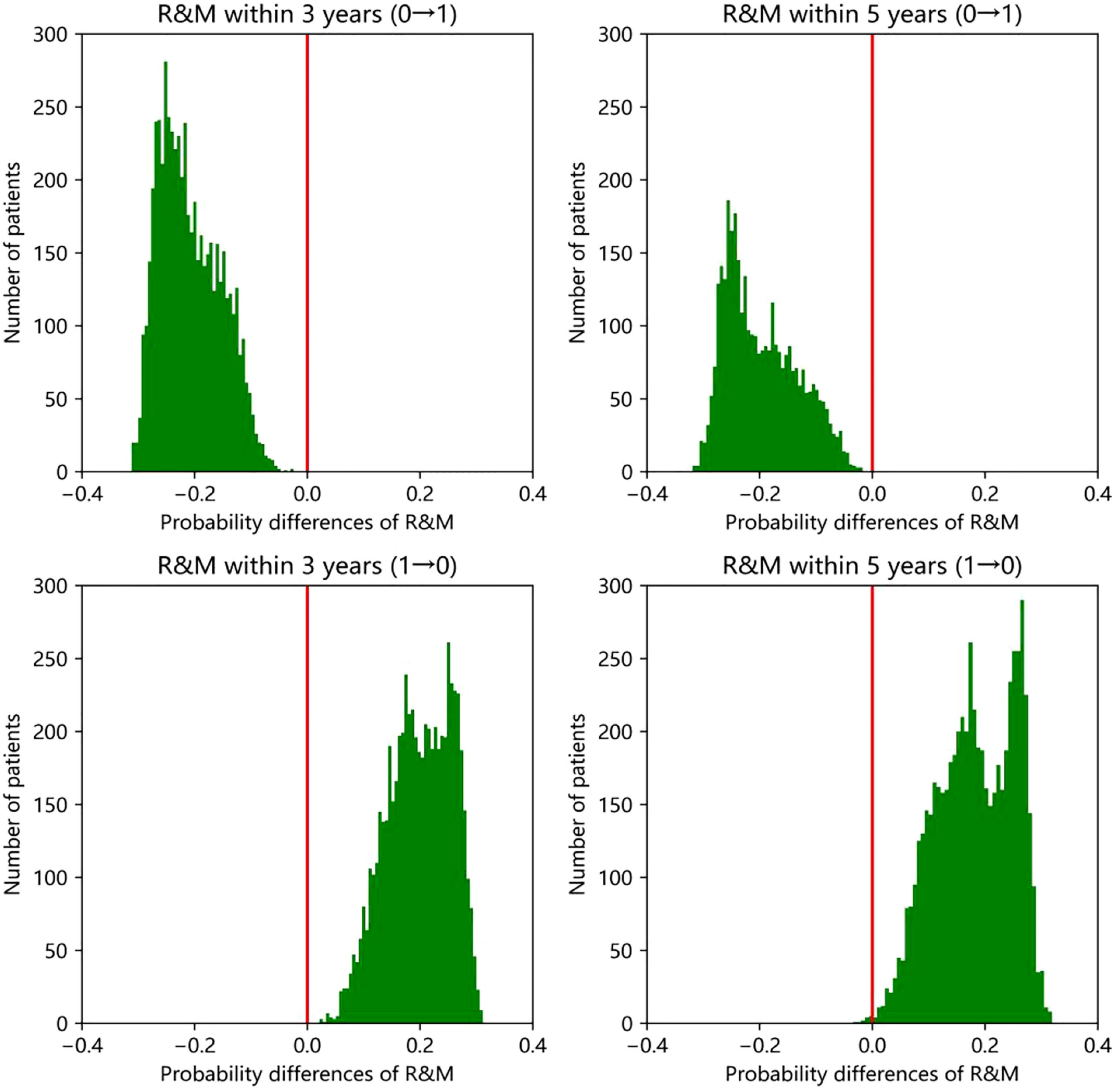
Effect of Chinese medicine on probability of R&M in XGBoost model.

Similarly, if the patients do not take Chinese medicine, the R&M rate will change. According to the prognosis of all patients in the statistical validation set, the prognostic model within 5 years constructed by the above XGBoost method can be obtained. If the patients who did not take Chinese medicine (a total of 61 patients), taking Chinese medicine reduced the R&M probability within 5 years by an average of 18 percentage points. Among the 34 patients taking Chinese medicine, there was an average increase of 18 percentage points in the R&M rate within 5 years if they did not take Chinese medicine. The degree of increase or decrease varied with individuals. In order to make the above results more statistically significant, that is, to reduce the accidental impact of the model or data set division on the research results, the data set was further randomly divided for 100 times to obtain the training set and validation set, and the model was trained and validated. Therefore, the change in R&M rate within 3 and 5 years of approximately several thousand person times can be obtained, including the change from taking Chinese medicine to not taking and vice versa. The number of patients with a change in R&M rate in different numerical ranges can be counted, and the histogram is shown in **Figure 6**. It can be seen from the figure that when all patients change from not taking Chinese medicine to taking Chinese medicine, the R&M rate decreased within 3 years or 5 years, with an average decrease of about 0.2. When changing from taking Chinese medicine to not taking Chinese medicine, the R&M rate in almost all patients increased within 3 years or 5 years, with an average increase of about 0.2.

1: Taking Chinese medicine; 0: Without Chinese medicine intervention

### Webpage deployment tool

**Figure 7:**
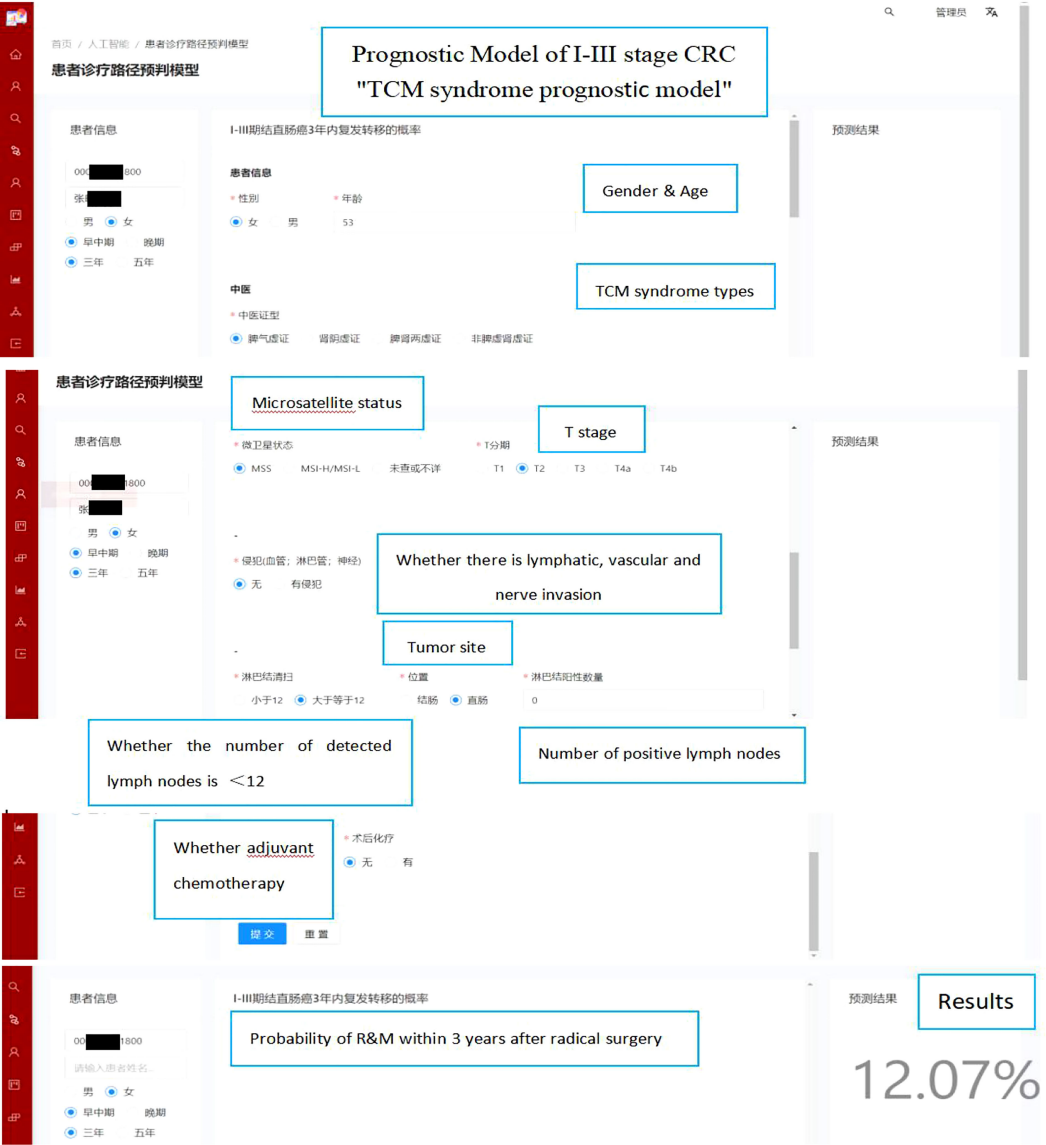
An example of the model visualizations.

We implemented our models into a website (http://www.xy.com) that provides risk probabilities for I-III stage CRC patients that can be used in the network within our two hospitals. As shown in **Figure 7**, baseline characteristics and disease information can be inputted on the left panel and estimated risks of R&M within 3 or 5 years are shown on the right panel. The source codes of our models are publicly available at https://gitee.com/doctortangmo/TCM-CRC-prognostic-model.

Using TCM syndrome prognostic model, an example of a 53-year-old female CRC patient is demonstrated on the webpage. The baseline characteristics and disease information are shown on the left panel, and the risks probability of R&M within 3 years is 12.07% that presented on the right panel.

## Discussion

CRC is a common malignant cancer, and its morbidity and mortality have increased significantly in recent years in China (43). Although the five-year survival rate of patients with early and middle stage CRC is more than 70% (44), R&M are the main reasons that threaten the long-term survival of patients. Approximately 30% to 50% of patients will have R&M (45, 46) and the five-year survival rate is less than 20% once they progress to an advanced stage (47). After receiving conventional treatment with Western medicine, patients with early and middle stage CRC entered the follow-up period. In China, about 80% of cancer patients will seek TCM treatment (48). Patients in the non-R&M stage of CRC choose TCM intervention as a complementary therapy, mainly to reduce clinical symptoms and reduce the probability of R&M (49).

In this study, we developed prognostic models with different TCM factors based on four different ML methods, LR, RF, XGBoost and SVM, which provided an objective estimate of the probability of R&M (0–100%) in patients in clinical practice. The basic theories of TCM hold that Shen (kidney) and Pi (spleen) are the origin of congenital constitution and acquired constitution, respectively. Cancer patients often have a declined autoimmune function caused by Shen (kidney) deficiency, and I-III stage CRC patients may have Pi (spleen) deficiency and residual disease after radical operation. Besides, several previous studies have pointed that I-III stage CRC patients can be treated with the “Jianpi-Bushen” rule (50, 51) during anti-R&M phase. In our study TCM syndrome types were included as predictors in constructing “TCM syndrome prognostic model”. This model can be used as an auxiliary tool to preliminarily explore the characteristics of the “beneficiary population” under integrated Chinese and Western medicine treatment.

Based on the RF and XGBoost methods, the “Chinese medicine intervention prognostic model” was constructed. Under the two prognostic scenarios of whether R&M occurred within 3 years and 5 years, the predictive ability of these models was equivalent, followed by SVM and LR. The AUC values of all models were above 0.75, showing good model discrimination. From the DCA curve, we found that the models constructed by the four methods had certain clinical utility. With regard to the “TCM syndrome prognostic model”, when predicting R&M within 3 years and 5 years, the model showed certain predictive ability and the AUC value of all models was above 0.70. Each of the four methods has its own advantages and disadvantages, but XGBoost was relatively better for model accuracy. XGBoost is an open-source ML method developed by Chen Tianqi and others in 2014, which is one of the boosting algorithms. It has the advantages of regularization promotion, parallel operation, autonomous learning and processing missing values (34). At present, it is well used in disease diagnosis, disease prognosis prediction and rational and safe drug use. However, when the sample size is large, the method consumes a large amount of memory and takes more time (52).

In addition to the AUC value commonly used to evaluate the model performance, the DCA curve was also selected as a clinical utility index of the model. This indicator was first developed by Professor Vickers of Memorial Sloan Kettering Cancer Center in 2008 (53). It can meet the actual needs of clinical decision-making. Therefore, the DCA curve is increasingly widely used in model development in the medical field (54). In this study, the DCA curve of the model constructed based on LR, RF and XGBoost had better clinical practicability and provided additional clinical benefits. Among them, the probability threshold of the RF method was the largest, demonstrating that it has the most extensive clinical reference value. Taking XGBoost as an example, this study described in detail how to use the model to quantify the risks probability of R&Mt and also gave a certain visual display. For the transparency and repeatability of our models, we have uploaded the relevant source codes on a public repository.

Several limitations need to be considered when interpreting our findings. Firstly, as the model to predict R&M involved CRC radical surgery and up to 5 years of follow-up, clinical outcome information acquisition, and some clinical features were missing; therefore, during the construction of the model, some factors as predictors of prognostic value were unable to be entered into the model. Secondly, retrospective case observation of some patients inevitably led to bias in the collection of clinical information. Thirdly, the model we established was based on data from only two hospitals where the source of anticipated individuals was relatively limited, and external validation of the model was temporarily unavailable. However, the above problems can be calibrated by expanding the patient validation set, enriching the data sources and using the prospective case observation method to improve the generalization ability and application of the model in the clinic. Last, interpretability and explainability of ML methods have become pressing issues, the blackbox nature of ML is still unresolved (55). It is of great significance to model application, especially in the field of medicine. Our study can still not be fully explained with the exact extent to which it can affect the model and impact the prediction outcomes. Thus we still advocate that clinicians and practitioners can approach these models with caution.

## Conclusions

In this study, CRC prognostic models were constructed based on different ML methods, and in general, these models showed good performance. The models can be used to predict the probability of R&M within 3 years and 5 years. Furthermore, based on the model, we can quantify the influence of “whether the patient accepts Chinese medicine intervention” and “different TCM syndromes” on the prognosis. However, it is still necessary to expand the sample size to calibrate the model and improve the generalization ability of the model through external validation sets. In conclusion, this study constructed R&M prognostic models containing TCM factors for the first time, and evaluated the model from the aspects of model discrimination and clinical utility. The models have good performance and can provide certain values for clinical decision-making.

## Data availability statement

The raw data supporting the conclusions of this article will be made available by the authors, without undue reservation.

## Ethics statement

Written informed consent was obtained from the individual(s) for the publication of any potentially identifiable images or data included in this article.

## Author contributions

All authors listed have made a substantial, direct, and intellectual contribution to the work, and approved it for publication.

## Funding

This study was supported by: 1. The National Administration of Traditional Chinese Medicine Inheriting and Innovating Traditional Chinese Medicine “Tens of Millions” Talent Project (Qihuang Project); 2. The National Natural Science Foundation of China (NSFC): “Study on the mechanism of tonifying spleen and fortifying kidney sequential treatment in improving chemotherapy-induced gastrointestinal and bone marrow toxicity *via* Wnt/β- catenin pathway mediated stem cell regeneration” (ID: 81973676).

## Acknowledgments

The authors appreciate all the patients and their families for making this study possible. We are grateful to the Computer Information Network departments in Xiyuan Hospital of Chinese Academy of Chinese Medical Sciences and Beijing Cancer Hospital for their collaboration in constructing a web page (www.xy.com) to embed our models.

## Conflict of interest

Author LG was employed by the company Smart City Business Unit, Baidu Inc., Beijing, China.

The remaining authors declare that the research was conducted in the absence of any commercial or financial relationships that could be construed as a potential conflict of interest.

## Publisher’s note

All claims expressed in this article are solely those of the authors and do not necessarily represent those of their affiliated organizations, or those of the publisher, the editors and the reviewers. Any product that may be evaluated in this article, or claim that may be made by its manufacturer, is not guaranteed or endorsed by the publisher.
