# Supplementary material for: Machine learning based prognostic model of Chinese medicine affecting the recurrence and metastasis of I-III stage colorectal cancer: A retrospective study in China
Source: Front Oncol. 2022 Nov 17;12:1044344. doi: 10.3389/fonc.2022.1044344 (PMC9714626; doi:10.3389/fonc.2022.1044344)
Supplement: Supplementary file 1 [file DataSheet_1.pdf]

Table 1. Performance Metrics of Chinese medicine intervention prognostic model (within 3 years)

	Methods			
Indicators	LR	RF	XGBoost	SVM
Precision	0.72 (0.63, 0.80)	0.73 (0.64, 0.82)	0.75 (0.68, 0.84)	0.67 (0.60, 0.74)
Recall	0.73 (0.60, 0.84)	0.77 (0.68, 0.86)	0.76 (0.66, 0.86)	0.85 (0.76,0.92)
Accuracy	0.76 (0.70, 0.83)	0.78 (0.72, 0.84)	0.79 (0.75, 0.84)	0.76 (0.70, 0.82)
F1 score	0.72 (0.62, 0.81)	0.74 (0.69, 0.80)	0.75 (0.69, 0.81)	0.75 (0.69, 0.79)

Table 2. Performance Metrics of Chinese medicine intervention prognostic model (within 5 years)

	Methods			
Indicators	LR	RF	XGBoost	SVM
Precision	0.79 (0.71, 0.87)	0.83 (0.75, 0.91)	0.84 (0.77, 0.93)	0.81 (0.72, 0.91)
Recall	0.66 (0.56, 0.76)	0.76 (0.67, 0.85)	0.75 (0.63, 0.85)	0.79 (0.70, 0.87)
Accuracy	0.70 (0.62, 0.79)	0.77 (0.70, 0.83)	0.78 (0.71, 0.83)	0.78 (0.71, 0.84)
F1 score	0.71 (0.63, 0.80)	0.79 (0.73, 0.85)	0.79 (0.72, 0.85)	0.82 (0.74, 0.86)

Table 3. Performance Metrics of TCM syndrome prognostic model (within 3 years)

	Methods			
Indicators	LR	RF	XGBoost	SVM
Precision	0.35 (0.25, 0.50)	0.36 (0.20, 0.57)	0.32 (0.21, 0.45)	0.29 (0.20, 0.42)
Recall	0.59 (0.33, 0.89)	0.42 (0.22, 0.67)	0.64 (0.33, 0.89)	0.54 (0.33,0.78)
Accuracy	0.76 (0.68, 0.84)	0.79 (0.72, 0.86)	0.72 (0.63, 0.82)	0.72 (0.65, 0.81)
F1 score	0.44 (0.31, 0.58)	0.38 (0.21, 0.57)	0.42 (0.26, 0.58)	0.37 (0.24, 0.58)

Table 4. Performance Metrics of TCM syndrome prognostic model (within 5 years)

	Methods			
Indicators	LR	RF	XGBoost	SVM
Precision	0.50	0.49	0.49	0.46

	(0.36, 0.67)	(0.33, 0.71)	(0.35, 0.67)	(0.33, 0.63)
Recall	0.59	0.51	0.72	0.59
	(0.33,0.78)	(0.22, 0.78)	(0.44, 1.00)	(0.33, 0.78)
Accuracy	0.74	0.73	0.73	0.71
	(0.66, 0.83)	(0.63, 0.83)	(0.63, 0.83)	(0.60, 0.80)
F1 score	0.53	0.49	0.58	0.52
	(0.35, 0.67)	(0.31, 0.70)	(0.46, 0.73)	(0.35, 0.67)
